# Supplementary material for: Detailed molecular and epigenetic characterization of the pig IPEC-J2 and chicken SL-29 cell lines
Source: iScience. 2023 Feb 20;26(3):106252. doi: 10.1016/j.isci.2023.106252 (PMC10018572; doi:10.1016/j.isci.2023.106252)
Supplement: Data S2. Complete homer output for identified motifs in Chicken SL-29, related to Tables 5 and 6 — Homer motif analysis results for histone modifications H3K4me1, H3K4me3, H3K27ac, enhancers, and ATAC-seq of chicken SL-29 cell line. Parameters for possible false positives is as mentioned earlier for S5. [file mmc3.zip › Data_S2/S6/Chicken_SL_29/peak_files_H3K4me1/homerResults/motif19.similar.html]

motif19

## Information for motif19

G
T
C
A
T
A
C
G
A
G
T
C
C
G
T
A
A
C
T
G
A
C
T
G
C
G
T
A
C
G
T
A
  
Reverse Opposite:  

A
C
G
T
A
C
G
T
A
G
T
C
A
G
T
C
A
C
G
T
A
C
T
G
A
T
G
C
A
C
G
T
  

|  |  |
| --- | --- |
| p-value: | 1e-26 |
| log p-value: | -6.016e+01 |
| Information Content per bp: | 1.909 |
| Number of Target Sequences with motif | 8721.0 |
| Percentage of Target Sequences with motif | 17.49% |
| Number of Background Sequences with motif | 7843.9 |
| Percentage of Background Sequences with motif | 15.72% |
| Average Position of motif in Targets | 148.7 +/- 86.2bp |
| Average Position of motif in Background | 150.5 +/- 87.6bp |
| Strand Bias (log2 ratio + to - strand density) | -0.0 |
| Multiplicity (# of sites on avg that occur together) | 1.10 |
| Motif File: | file (matrix) reverse opposite |

### Similar de novo motifs found

|  |  |  |  |  |  |  |  |
| --- | --- | --- | --- | --- | --- | --- | --- |
| Rank | Match Score | Redundant Motif | P-value | log P-value | % of Targets | % of Background | Motif file |
| 1 | 0.640 | A G C T C A G T A C T G G A C T A G C T A G C T A G T C G A T C G C A T A C T G G C T A G T A C A C T G | 1e-24 | -55.755062 | 0.05% | 0.00% | motif file (matrix) |
| 2 | 0.646 | T G A C G C T A T C A G G T A C C T A G C A T G C A T G T G C A C T G A G T A C G C T A A C G T G A C T | 1e-23 | -53.101054 | 0.06% | 0.01% | motif file (matrix) |
| 3 | 0.641 | C G A T A C T G A T C G C G A T A C G T C G A T A G T C A C G T C G A T C T A G | 1e-20 | -47.206840 | 2.88% | 2.23% | motif file (matrix) |
